# Atypical Wassel type VI thumb duplication treated with on-top plasty

**DOI:** 10.1016/j.jpra.2023.12.007

**Published:** 2023-12-12

**Authors:** Krešimir Bulić, Goran Tintor

**Affiliations:** a Department of Surgery, University Hospital Centre Zagreb, University of Zagreb School of Medicine, Zagreb, Croatia, Kišpatićeva 12, 10000 Zagreb, Croatia; b Deparment of Surgery, University Hospital Centre Split, Spinčićeva 1, 21000 Split, Croatia

**Keywords:** Child, On-top plasty, Surgical procedures, Thumb duplication, Treatment outcome, Wassel type VI

## Abstract

Congenital thumb duplication is estimated to occur between 0.08 and 7.6 times per 1,000 live births; however its cause is still undetermined. In this report, we present a case of Wassel type VI thumb polydactyly. clinical examination revealed an optimal functional position and an aesthetically pleasing shape of the ulnar thumb as well as a superior nail and pulp. However, preoperative X-ray indicated a well formed carpometacarpal joint of the radial thumb compared to an underdeveloped CMC joint of the ulnar thumb. Through surgical procedure we combined the best parts of both thumbs with on-top plasty to achieve the most optimal outcome. In conclusion, it is important to determine an adequate treatment strategy for a patient based on both clinical and radiological assessments.

Congenital thumb duplication is estimated to occur between 0.08 and 7.6 times per 1,000 live births; however its cause is still undetermined.^1^, ^2^, ^3^ Furthermore,only a few studies have objectively and quantitatively examined the outcomes of surgical treatment.^4^^,^^5^ The most widely used Wassel classification of thumb duplications in seven types is practical and based on the level of duplication, but it doesn't provide a knowledge of soft-tissue anatomy, stability and mobility of the joints or which thumb possess appropriate size and shape.^6^

In this report, we present a case of Wassel type VI thumb polydactyly. During the assessment of the patient's condition, clinical examination revealed an optimal functional position and an aesthetically pleasing shape of the ulnar thumb as well as a superior nail and pulp. However, preoperative X-ray indicated a well formed carpometacarpal joint of the radial thumb compared to an underdeveloped CMC joint of the ulnar thumb (Figure 1).

**Figure 1:**
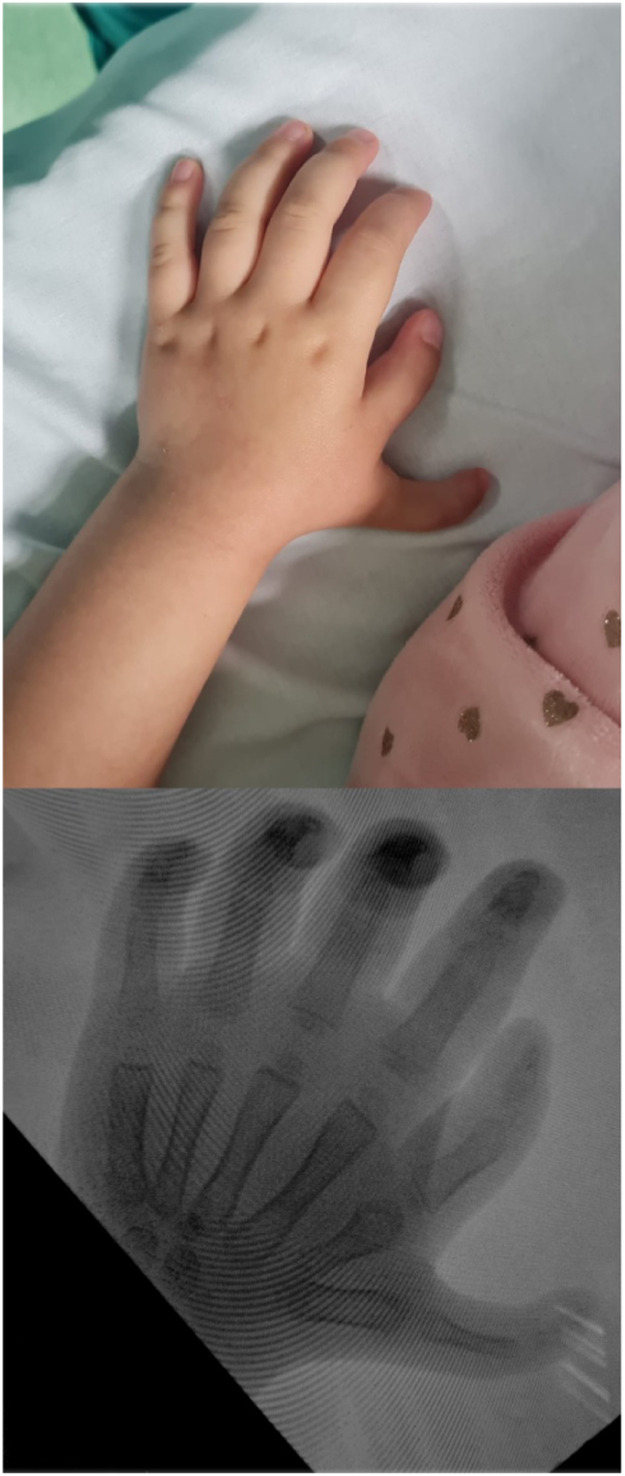
Wassel VI of the thumb is evident on a preoperative plain radiograph.

Following the excision of the skin and subcutaneous tissue around the radial thumb, dissection was extended up to the metacarpophalangeal joint. The abductor pollicis brevis (APB) and extensor pollicis longus (EPL) tendons were divided at their insertions. An amputation of the radial thumb was performed at the level of the lower third of the metacarpal bone to preserve the capsular-periosteal attachment of the CMC joint. A transverse osteotomy of the ulnar thumb was performed to remove articular surface of the metacarpal base and the distal part of the ulnar thumb was transposed onto the proximal part of the radial thumb. The bones were fixed with a longitudinal K-wire and the reconstruction was completed with a transposition of the APB and EPL tendons to the newly created insertions on the CMC joint and extensor apparatus (Figure 2).

**Figure 2:**
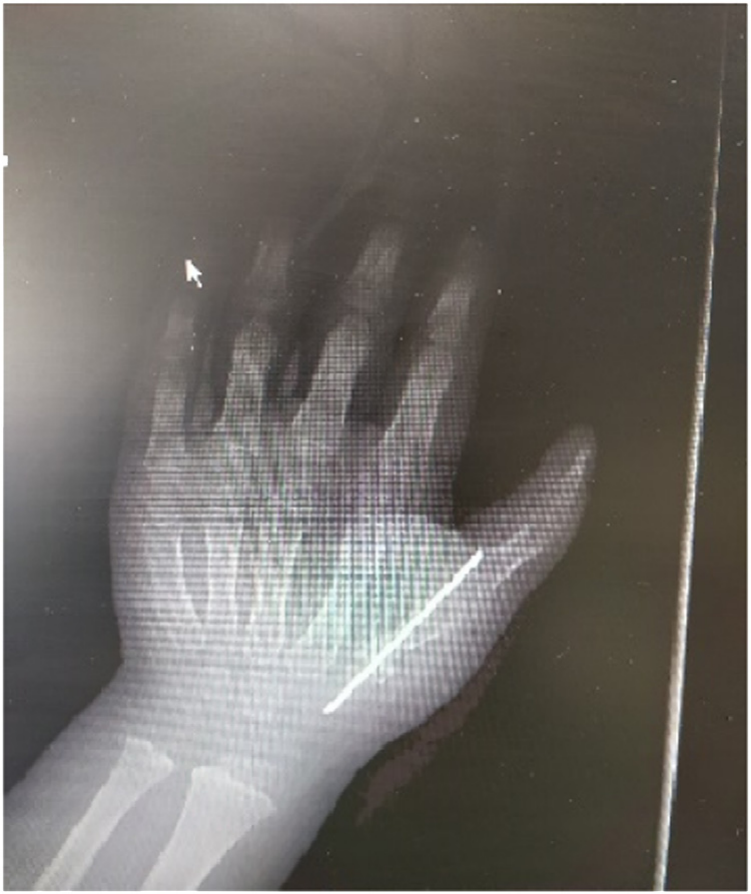
Intraoperative image after fixation of the remaining bone fragments with Kirschner wire.

Finally, the excision was closed with an absorbable suture. A long-arm splint was applied for 6 weeks, after which time the K-wire was removed. Although the option of excising the rudimentary accessory phalanx causing a 15-degree ulnar deviation was presented to the parents, they decided not to have it performed at this time due to a fully functional and aesthetically pleasing thumb (Figure 3).

**Figure 3:**
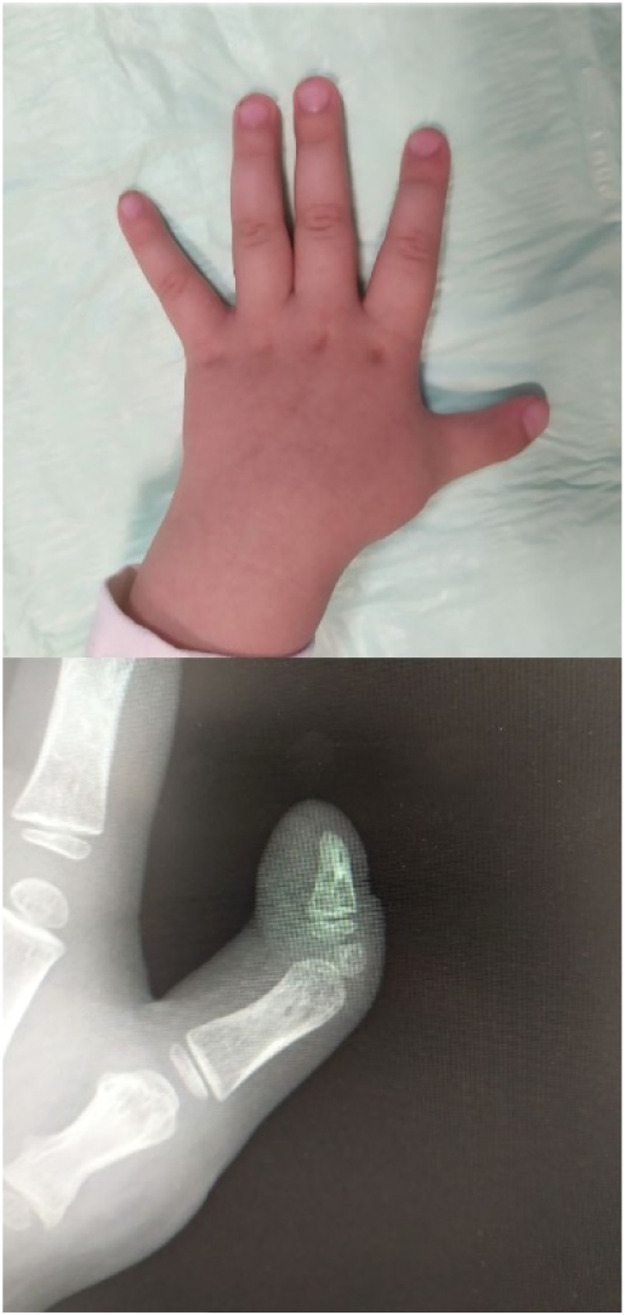
Images taken six weeks after surgery exhibiting the thenar eminence's contour and the adequate CMC joint of the thumb.

The majority of research on surgical interventions for thumb duplication is focused on the two most prevalent forms, the Wassel II and Wassel IV. However, the incidence and optimal treatment of infrequent variants are not as well documented. Shen et al. demonstrated their on-top reconstructive procedure for Wassel VI radial polydactyly with triphalangeal thumb deformity. The study concentrated on osseous restructuring and simplified surgical technique without taking into consideration the soft-tissue reconstruction due to an uncommon and challenging variant – a mixture of Wassel VI and Wassel VII subtypes.^7^ In our case report, we incorporated the description of the surgical approach, which puts emphasis not just on the bone reconstruction but also on the tendon transfer and capsular-periosteal flap preservation.

Ogino et al. revealed that insufficient results have been prevalent in Wassel types III, V, and VI, as well as triphalangeal-type thumb polydactyly. This outcome was primarily attributed to the adduction contracture of the thumb.^8^ In contrast to these findings, our case demonstrated a cosmetically pleasing postoperative result as well as the maintenance of optimal function.

In conclusion, it is important to determine an adequate treatment strategy for a patient based on both clinical and radiological assessments.

## Declaration of Competing Interest

None declared.
